# Sex Differences in Cardiovascular Disease Risk and Associated Risk Factors among People Living with HIV in China

**DOI:** 10.5334/gh.1553

**Published:** 2026-04-24

**Authors:** Junwen Yu, Xiaoning Liu, Yun He, Zheng Zhu, Yuanyuan Xu, Changrong Yuan, Hongzhou Lu

**Affiliations:** 1 School of Nursing, Fudan University, Shanghai, China; 2 Department of Infectious Diseases, National Clinical Research Center for Infectious Diseases, the Third People’s Hospital of Shenzhen, Guangdong, China; 3 Fudan University Centre for Evidence-based Nursing: A Joanna Briggs Institute Centre of Excellence, Shanghai, China

**Keywords:** Cardiovascular disease, Cardiovascular risk factor, HIV, Sex differences

## Abstract

**Background:**

People living with HIV (PLWH) have twice the risk of developing cardiovascular disease (CVD) compared to HIV-negative individuals, yet sex differences remain unclear. This study aimed to address this gap by examining sex differences in CVD risk among Chinese PLWH.

**Methods:**

We analyzed data from hospitalized PLWH in the Third People’s Hospital of Shenzhen from 2017 to 2022. The 10-year CVD risk was estimated using the pooled cohort equations. Logistic regression and predicted marginal analyses were performed to assess adjusted sex differences in CVD risk factors. An exploratory segmented trend analysis based on five-year age groups was conducted to examine the relationship between age and the prevalence of high CVD risk.

**Results:**

Among the 3020 participants, 7.7% had high CVD risk. Men living with HIV had a significantly higher predicted probability of high CVD risk compared with women (9.3% vs. 2.1%, *P* < 0.001). Among PLWH aged 18–49 years, men were significantly more likely to exhibit several CVD risk factors, including decreased high-density lipoprotein cholesterol (65.6% vs. 48.5%, *P* < 0.001), overweight/obesity (17.7% vs. 11.2%, *P* < 0.010), diabetes mellitus (3.0% vs. 1.2%, *P* < 0.026), and declined estimated glomerular filtration rate (13.8% vs. 8.4%, *P* < 0.005).

**Conclusion:**

Significant sex disparities were observed in the predicted CVD risk and major risk factors. Healthcare providers should provide early and sex-responsive interventions, particularly for younger males with multiple modifiable risk factors and for females, in whom risk may rise rapidly postmenopause. These findings support the need for equitable, tailored strategies in CVD risk assessment and prevention for PLWH.

## Introduction

The global expansion of effective antiretroviral therapy (ART) has transformed HIV from a fatal disease into a manageable chronic condition (1). People living with HIV (PLWH) can now expect to live a near-normal life span. However, this success has created a new challenge: PLWH are twice as likely to develop cardiovascular disease (CVD) compared to HIV-negative individuals and experience a three-fold increase in CVD-related disability-adjusted life years (2,3). The elevated CVD burden is attributable to both traditional risk factors and HIV-specific factors. Previous studies found that PLWH have a higher prevalence of traditional CVD risk factors, such as hypertension, dyslipidemia, and smoking than the general population (4). Notably, CVD remains highly prevalent among PLWH even in the absence of these traditional risk factors (5), underscoring the contribution of HIV-specific mechanisms, including chronic inflammation, persistent immune activation, and long-term exposure to ART (6,7). Therefore, the focus of research and clinical care is shifting towards the screening, prevention, and management of CVD as a crucial component of long-term care for PLWH.

While CVD has become a growing concern among PLWH, accumulating evidence indicates significant sex differences in both its incidence and mortality (8). Previous study found that men have a higher incidence of atherosclerotic cardiovascular disease (ASCVD) than women among PLWH (9). However, compared to HIV-negative populations, the increased prevalence of cardiovascular events observed among PLWH is more pronounced in women, suggesting that the increased risk of CVD conferred by HIV may be even higher in women (8). Additionally, HIV appears to elevate the risk of CVD-related mortality more significantly in women than in men (10). Together, these disparities in CVD incidence and outcomes imply that the underlying risk factors contributing to cardiovascular risk may also differ by sex. Understanding the sex-specific risk profiles is essential for developing targeted CVD prevention and management strategies for both men and women living with HIV.

Although previous studies have examined sex differences in the prevalence of CVD risk factors, findings were inconsistent. For example, studies reported a higher prevalence of diabetes mellitus (DM) among women with HIV (11), whereas others found the opposite result (12). Such inconsistencies make it difficult to draw clear conclusions about the direction and magnitude of sex disparities. Additionally, most existing research comes from Western countries (8). However, the HIV epidemic pattern, distribution of CVD risk factors, socioeconomic context, and ART regimens in China differ substantially from those in Western settings. As a result, sex-specific findings from prior studies cannot be directly extrapolated to the Chinese PLWH population. There is an urgent need to characterize the CVD risk factor profile and its sex differences among PLWH in China to inform tailored primary prevention strategies. Moreover, few studies have focused on sex differences in 10-year predicted CVD risk. Examining predicted risk, rather than clinical events such as incidence or mortality, allows earlier identification of high-risk groups and carries important implications for prevention. Nevertheless, it remains unclear whether, and to what extent, sex differences in both predicted CVD risk and major CVD risk factors exist among PLWH in China.

Therefore, the aim of our study was to explore the sex differences in CVD risk and associated risk factors among PLWH in China. Understanding these differences provides novel and context-specific evidence to develop tailored prevention that addresses the unique cardiovascular health needs of both men and women living with HIV.

## Methods

### Study design and setting

This cross-sectional study used electronic health record (EHR) data at the Third People’s Hospital of Shenzhen between September 1, 2017, and September 30, 2022. The Third People’s Hospital of Shenzhen is the only designated hospital for HIV care in Shenzhen. The sample that was observed in our study was representative of the characteristics of PLWH in the southeastern region of China. The study was approved by the Research Ethics Committee of the hospital (registration number: 2024–008).

### Participants

We included participants if they (i) were diagnosed with HIV-1; (ii) were aged 18 years or older; (iii) were hospitalized between September 1, 2017, and September 30, 2022. Patients with any prior CVD, including coronary heart disease, myocardial infarction, stroke, angina, and heart failure, were excluded. Meanwhile, individuals without risk factor data for the pooled cohort equation (PCE) estimation were excluded. PCE risk factor data including age, total cholesterol (TC), high-density lipoprotein cholesterol (HDL-C), systolic blood pressure (SBP), smoking, and DM.

### Measurements

#### Cardiovascular disease risk assessment

The PCE was used to estimate individuals’ 10-year CVD risk. It was originally developed for White and African American populations in the United States (13) and has been widely applied in CVD risk assessment among PLWH because it provides a standardized, clinically interpretable framework for risk stratification based on routinely available variables. As no CVD risk prediction model has been developed for or validated in Chinese PLWH, the PCE offered a practical approach for risk estimation in this cohort. The predictive outcome of CVD was the first hard ASCVD event, defined as nonfatal MI, CHD death, and fatal or nonfatal stroke. Risk levels were stratified as low (<7.5%) and high (≥7.5%). Previous studies found that the discrimination for applying PCE in PLWH was acceptable with a C-statistic of 0.72 (95% CI, 0.67–0.76) (14).

#### Sociodemographic variables

We collected the self-reported sociodemographic data from the EHR, including age (continuous), sex (male and female), birthplace (South, North, and unknown), race/ethnicity (Han and minority), employment status (employed and unemployed), marital status (married, single, divorced, widowed, and otherwise), and health insurance coverage (any private or public health insurance and self-paying).

#### Cardiovascular disease risk variables

Traditional risk factors included overweight (body mass index [BMI] 24–27 kg/m^2^) and obesity (BMI≥28 kg/m^2^), elevated SBP (≥120 mmHg), current smoking (yes or no), current alcohol consumption (yes or no), dyslipidemia (TC≥5.2 mmol/L, HDL-C < 1 mmol/L, low-density lipoprotein cholesterol [LDL]≥3.4 mmol/L, or triglyceride [TG]≥1.7 mmol/L) (15), declined estimated glomerular filtration rate (eGFR) (<90 ml/min/1.73 m^2^), hypertension (clinical diagnostic records or prescription of antihypertensive drugs), DM (clinical diagnostic records), CRP (C-reactive protein), and D-dimer. HIV-related risk factors included low CD4 count (<200 cells/μL), CD8 (continuous), and HIV viral suppression (plasma HIV-1 RNA<500 copies/ml).

### Statistical analysis

Statistical analyses were conducted using R software 4.3.2 and Stata 17.0. We calculated means, standard deviations, medians, interquartile ranges (IQRs), frequencies, and percentages to describe the characteristics of men and women in both age groups, 18–49 and ≥50 years. Comparisons between groups were performed using Student’s t-test and Mann-Whitney U test for continuous variables, and the chi-square test for categorical variables.

Multivariate logistic regression was used to identify factors associated with high CVD risk (PCE score≥7.5%). Predicted marginal analysis was subsequently used to estimate the predicted probabilities and prevalence differences of CVD risk factors between men and women within each age group. Potential explanatory variables were introduced in a stepwise manner. Model 1 was unadjusted, with sex as the independent variable and the CVD risk factor as the dependent variable. Model 2 was adjusted for sociodemographic variables, including age, birthplace, employment status, marital status, and health insurance coverage. Model 3 further adjusted for physiological and behavioral variables associated with CVD risk factors.

We further examined whether sex modified the associations between individual cardiometabolic risk factors and high predicted CVD risk by including sex-by-risk factor interaction terms in logistic regression models. Sex-stratified models were also fitted separately for men and women.

An exploratory segmented trend analysis based on five-year age groups was conducted to examine the relationship between age and the prevalence of high CVD risk. For each five-year interval, we calculated the age-specific prevalence of high CVD risk and fitted two-piecewise linear regression models to characterize changes in trend. The optimal breakpoint was selected using the Akaike information criterion (AIC). Results were plotted separately for men and women. To assess breakpoint uncertainty, age was alternatively grouped into 10-year intervals, and the age-specific prevalence of high CVD risk was replotted to evaluate whether the trend and the approximate age range of risk increase remained consistent.

## Results

### Demographic and clinical characteristics

**Figure 1:**
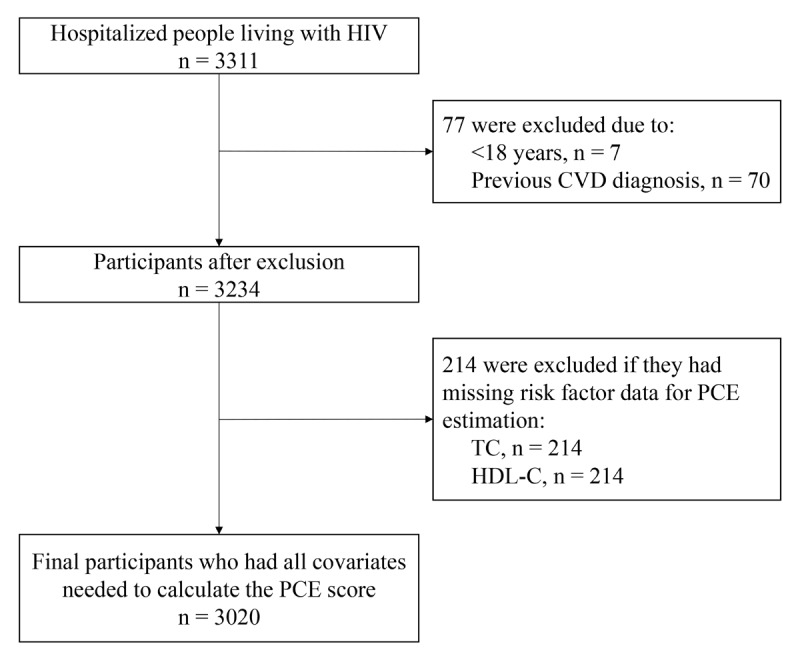
Flow diagram of the selection process.

Between September 1, 2017, and September 30, 2022, a total of 3311 PLWH were hospitalized and screened for the study. After excluding 70 individuals with a history of CVD, 7 under the age of 18, and 214 who lacked the risk factor data for PCE estimation, 3020 participants were included in our analysis (Figure 1).

The characteristics of the included participants are detailed in Table 1. Among the 3020 participants, 2703 (89.5%) were males, 2444 (80.9%) were under the age of 50, 2144 (71%) were currently employed, 1577 (52.2%) were single, and 2493 (82.5%) had health insurance coverage. The median CD4 count was 238 (IQR, 67–483) cells/mm^3^.

**Table 1:** Characteristics of the included participants (n = 3020).


	TOTAL (n = 3020)	AGE 18–49 YEARS (n = 2444)			AGE ≥50 YEARS (n = 576)		
	
		FEMALE (n = 203)	MALE (n = 2241)	*P* VALUE	FEMALE (n = 114)	MALE (n = 462)	*P* VALUE

Age, mean ± SD, years	39.29 ± 11.99	37.39 ± 7.44	34.50 ± 7.43	<0.001	58.82 ± 6.58	58.51 ± 7.80	0.694

18–49, n(%), years	2444 (80.9)						

≥50, n(%), years	576 (19.1)						

Male, n(%)	2703 (89.5)						

Nation, Han, n(%)	2895 (95.9)	193 (95.1)	2143 (95.6)	0.714	110 (96.5)	449 (97.2)	0.695

Birthplace, n(%)				<0.001			0.075

South	2471 (81.8)	184 (90.6)	1795 (80.1)		104 (91.2)	388 (84.0)	

North	509 (16.9)	14 (6.9)	418 (18.7)		8 (7.0)	69 (14.9)	

Unknown	40 (1.3)	5 (2.5)	28 (1.2)		2 (1.8)	5 (1.1)	

Employed, n(%)	2144 (71)	131 (64.5)	1754 (78.3)	<0.001	31 (28.2)	228 (49.4)	<0.001*

Marital status, n(%)				<0.001			<0.001*

Married	1163 (38.5)	139 (68.5)	570 (25.4)		87 (76.3)	367 (79.4)	

Single	1577 (52.2)	34 (16.7)	1514 (67.6)		1 (0.9)	28 (6.1)	

Divorce	203 (6.7)	22 (10.8)	129 (5.8)		6 (5.3)	46 (10.0)	

Widowed	55 (1.8)	7 (3.4)	9 (0.4)		20 (17.5)	19 (4.1)	

Otherwise	22 (0.7)	1 (0.5)	19 (0.8)		0 (0.0)	2 (0.4)	

Health insurance coverage, n(%)	2493 (82.5)	162 (79.8)	1913 (85.4)	0.034	72 (63.2)	346 (74.9)	0.012*

BMI, mean ± SD, kg/m^2^	21.46 ± 3.17	20.63 ± 2.76	21.51 ± 3.22	<0.001	21.32 ± 3.27	21.65 ± 3.00	0.304

<18.5, n(%), kg/m^2^	496 (16.4)	51 (25.1)	366 (16.3)	<0.001	21 (18.4)	58 (12.6)	0.197

18.5–23.9, n(%), kg/m^2^	1991 (65.9)	133 (65.5)	1474 (65.8)		69 (60.5)	315 (68.5)	

≥24, n(%), kg/m^2^	533 (17.6)	19 (9.4)	401 (17.9)		24 (21.1)	89 (19.3)	

SBP, mean ± SD, mmHg	108.96 ± 8.63	108.61 ± 8.03	109.09 ± 8.69	0.449	109.04 ± 9.30	108.47 ± 8.46	0.534

<120, n(%), mmHg	2660 (88.1)	180 (88.7)	1966 (87.7)	0.695	99 (86.8)	415 (89.9)	0.357

≥120, n(%), mmHg	360 (11.9)	23 (11.3)	275 (12.3)		15 (13.2)	47 (10.2)	

Hypertension, n(%)	208 (6.9)	9 (4.4)	93 (4.1)	0.847	18 (15.8)	88 (19.0)	0.421

DM, n(%)	140 (4.6)	3 (1.5)	66 (2.9)	0.227	15 (13.2)	56 (12.1)	0.763

Smoking, n(%)	424 (14)	7 (3.4)	333 (14.9)	<0.001	0 (0.0)	84 (18.2)	<0.001*

Alcohol consumption, n(%)	93 (3.1)	1 (0.5)	67 (3.0)	0.038	0 (0.0)	25 (5.4)	0.011*

HIV-RNA≥500 copies/ml, n(%)	1127 (37.3)	77 (37.9)	829 (37.0)	0.791	43 (37.7)	178 (38.5)	0.874

CD4 count <200 cells/μL, n(%)	1269 (42)	87 (42.9)	926 (41.3)	0.67	40 (35.1)	216 (46.8)	0.025*

CD4/CD8 ratio, mean ± SD	0.46 ± 0.41	0.53 ± 0.47	0.44 ± 0.39	0.012	0.51 ± 0.39	0.48 ± 0.47	0.487

eGFR, mean ± SD, ml/min/1.73 m^2^	105.36 ± 23.66	111.52 ± 18.23	109.81 ± 21.20	0.208	84.48 ± 24.20	86.20 ± 24.58	0.501

≥90, n(%), ml/min/1.73 m^2^	2439 (80.8)	182 (89.7)	1937 (86.4)	0.196	61 (53.5)	259 (56.1)	0.623

<90, n(%), ml/min/1.73 m^2^	581 (19.2)	21 (10.3)	604 (13.6)		53 (46.5)	203 (43.9)	

TC, mean ± SD, mmol/L	3.84 ± 1.08	4.02 ± 1.20	3.78 ± 1.05	0.006	4.22 ± 1.28	3.94 ± 1.09	0.016*

<5.2, n(%), mmol/L	2720 (90.1)	173 (85.2)	2044 (91.2)	0.005	95 (83.3)	408 (88.3)	0.152

≥5.2, n(%), mmol/L	300 (9.9)	30 (14.8)	197 (8.8)		19 (16.7)	54 (11.7)	

HDL-C, mean ± SD, mmol/L	0.91 ± 0.33	1.07 ± 0.41	0.88 ± 0.31	<0.001	1.04 ± 0.36	0.92 ± 0.33	<0.001*

>1, n(%), mmol/L	1107 (36.7)	108 (53.2)	769 (34.3)	<0.001	57 (50.0)	173 (37.4)	0.014*

≤1, n(%), mmol/L	1913 (63.3)	95 (46.8)	1472 (65.7)		57 (50.0)	289 (62.6)	

LDL-C, mean ± SD, mmol/L	2.31 ± 0.81	2.34 ± 0.86	2.29 ± 0.79	0.432	2.49 ± 0.95	2.37 ± 0.82	0.180

<3.4, n(%), mmol/L	2771 (91.8)	181 (89.2)	2072 (92.5)	0.094	101 (88.6)	417 (90.3)	0.597

≥3.4, n(%), mmol/L	249 (8.2)	22 (10.8)	169 (7.5)		13 (11.4)	45 (9.7)	

TG, mean ± SD, mmol/L	1.67 ± 1.12	1.66 ± 1.10	1.67 ± 1.11	0.901	1.73 ± 1.11	1.67 ± 1.18	0.633

<1.7, n(%), mmol/L	2003 (66.3)	135 (66.5)	1492 (66.6)	0.983	72 (63.2)	304 (65.8)	0.596

≥1.7, n(%), mmol/L	1017 (33.7)	68 (33.5)	749 (33.4)		42 (36.8)	158 (34.2)	

Any form of dyslipidemia, n(%)	2312 (76.6)	138 (68.0)	1730 (77.2)	0.003	81 (71.1)	363 (78.6)	0.087

CRP, median (IQR), mg/L	5.94 (24.16)	3.66 (16.42)	5.60 (24.15)	0.004	6.75 (24.46)	9.82 (32.32)	0.028*

D-dimer, median (IQR), mg/L	0.65 (0.25, 1.18)	0.83 (0.9)	0.57 (0.94)	0.034	1.15 (0.85)	1.11 (0.85)	0.647

PCE score, mean ± SD, %	2.27 ± 4.26	0.90 ± 1.58	0.98 ± 1.50	0.46	3.58 ± 2.97	8.79 ± 7.26	<0.001*

<5%, n(%)	2647 (87.6)	199 (98.0)	2186 (97.5)	0.365	87 (76.3)	175 (37.9)	<0.001*

5–7.4%, n(%)	139 (4.6)	1 (0.5)	38 (1.7)		16 (14.0)	84 (18.2)	

7.5–19.9%, n(%)	202 (6.7)	3 (1.5)	16 (0.7)		11 (9.6)	172 (37.2)	

≥20%, n(%)	32 (1.1)	0 (0.0)	1 (<0.01)		0 (0.0)	31 (6.7)	


Abbreviation: BMI: body mass index, CRP: C-reactive protein, DM: diabetes mellitus, eGFR: estimated glomerular filtration rate, HDL-C: high-density lipoprotein cholesterol, IQR: interquartile ranges, LDL-C: low-density lipoprotein cholesterol, PCE: the pooled cohort equation, SBP: systolic blood pressure, SD: standard deviation, TC: total cholesterol, TG: triglyceride. *P < 0.05.

### Cardiovascular risk and prevalence of traditional risk factors

The average 10-year CVD risk among the participants was 2.27 ± 4.26%, with 234 (7.7%) participants stratified as having a high risk (Table 1). Dyslipidemia was the most common cardiometabolic abnormality (76.6%), driven largely by the high prevalence of low HDL-C (63.3%).

Other traditional risk factors such as hypertension (6.9%), DM (4.6%), and current alcohol consumption (3.1%) were relatively less common.

### Risk factors associated with PCE score

Table 2 shows the risk factors associated with PCE score among PLWH. Being male (*OR* [*95% CI*] = 9.78 [5.10–18.74], *P* < 0.001), older (≥50 years) (*OR* [*95% CI*] = 31.39 [18.21–54.11], *P* < 0.001), unemployed (*OR* [*95% CI*] = 0.29 [0.20–0.43], *P* < 0.001), married (*OR* [*95% CI*] = 0.24 [0.12–0.49], *P* < 0.001), current alcohol consumption (*OR* [*95% CI*] = 6.05 [2.59–14.11], *P* < 0.001), unsuppressed HIV viral load (*OR* [*95% CI*] = 1.74 [1.14–2.65], *P* < 0.011), declined eGFR (*OR* [*95% CI*] = 2.41 [1.66–3.50], *P* < 0.001), and elevated TG (*OR* [*95% CI*] = 1.56 [1.07–2.28], *P* < 0.021) were significantly associated with a high risk of CVD.

**Table 2:** Factors associated with a high cardiovascular disease risk in multivariable logistic regression model.


FACTOR	*ODD RATIO*	*95% CI*	*P* VALUE

Age ≥50 years	31.39	(18.21–54.11)	<0.001*

Male	9.78	(5.10–18.74)	<0.001*

Birthplace South	0.93	(0.56–1.54)	0.780

Employed	0.29	(0.20–0.43)	<0.001*

Single (compared to married)	0.24	(0.12–0.49)	<0.001*

Health insurance coverage	0.88	(0.58–1.33)	0.534

Alcohol consumption	6.05	(2.59–14.11)	<0.001*

Overweight/Obesity	1.04	(0.65–1.64)	0.884

HIV-RNA≥500 copies/ml	1.74	(1.14–2.65)	0.011*

CD4 count <200 cells/μL	0.82	(0.54–1.24)	0.336

Decline eGFR	2.41	(1.66–3.50)	<0.001*

High LDL-C	1.73	(0.96–3.11)	0.069

High TG	1.56	(1.07–2.28)	0.021*

CRP	1.00	(1.00–1.01)	0.505

D-DIC	0.98	(0.88–1.08)	0.627


Abbreviation: CRP: C-reactive protein, eGFR: estimated glomerular filtration rate, LDL-C: low-density lipoprotein cholesterol, TG: triglyceride. *P < 0.05.

No significant sex-by-risk factor interactions were observed (all *P* > 0.05). Detail results of the interaction analyses and sex-stratified analyses are presented in Supplementary Table S1 and Table S2.

### Sex differences in CVD risk and risk factors

Table 3 shows sex differences of the adjusted predicted probability in CVD risk factors, with detailed information provided in Supplementary Table S3. The predicted marginal probability of being at high CVD risk was 9.3% (8.5–10.2%) for males and 2.1% (1.1–3.1%) for females (*P* < 0.001).

**Table 3:** Sex differences of the adjusted predicted probability in CVD risk factors.


	FEMALE (%)	MALE (%)	PREVALENCE DIFFERENCE (%)	*P* VALUE

**Age 18–49 years (n = 2444)**				

High CVD risk	1.6 (–0.3–3.5)	0.8 (0.4–1.1)	–0.9	0.372

High TC	13.6 (8.8–18.4)	8.9 (7.7–10.0)	–4.8	0.060

Low HDL-C	48.5 (41.9–55.1)	65.5 (63.7–67.4)	17.0	<0.001*

High LDL-C	10.8 (6.3–15.3)	7.5 (6.5–8.6)	–3.2	0.175

High TG	31.8 (25.4–38.3)	33.6 (31.7–35.5)	1.7	0.613

Overweight/obesity	11.2 (6.5–15.8)	17.7 (16.2–19.2)	6.5	0.010*

DM	1.2 (–0.2–2.6)	3.0 (2.3–3.7)	1.8	0.026*

SBP elevated	13.1 (7.9–18.3)	12.1 (10.8–13.5)	–1.0	0.728

Declined eGFR	8.4 (4.9–11.9)	13.8 (12.4–15.2)	5.4	0.005*

HIV-RNA≥500 copies/ml	35.8 (30.1–41.5)	37.2 (35.5–38.8)	1.4	0.649

CD4 count <200 cells/μL	40.5 (34.4–46.5)	41.5 (39.8–43.3)	1.1	0.736

**Age ≥50 years (n = 576)**				

High CVD risk	11.6 (7.6–15.7)	44.7 (41.6–47.8)	33.1	<0.001*

High TC	15.2 (8.6–21.8)	12.0 (9.1–15.0)	–3.1	0.411

Low HDL-C	55.7 (46.6–64.8)	61.0 (56.7–65.3)	5.3	0.314

High LDL-C	12.1 (5.6–18.5)	9.6 (7.0–12.3)	–2.4	0.502

High TG	38.2 (28.9–47.5)	33.6 (29.3–37.8)	–4.6	0.388

Overweight/obesity	23.0 (14.8–31.2)	18.9 (15.4–22.4)	–4.1	0.377

DM	12.3 (6.1–18.4)	12.4 (9.4–15.4)	0.1	0.968

SBP elevated	14.9 (7.6–22.2)	9.9 (7.2–12.6)	–5.0	0.216

Declined eGFR	45.7 (36.5–54.9)	44.1 (39.8–48.4)	–1.5	0.772

HIV-RNA≥500 copies/ml	41.8 (34.2–49.5)	37.5 (33.9–41.1)	–4.3	0.328

CD4 count <200 cells/μL	35.5 (27.5–43.6)	46.8 (42.8–50.9)	11.3	0.016*


Abbreviation: DM: diabetes mellitus, eGFR: estimated glomerular filtration rate, HDL-C: high-density lipoprotein cholesterol, LDL-C: low-density lipoprotein cholesterol, SBP: systolic blood pressure, TC: total cholesterol, TG: triglyceride. This model was adjusted for sociodemographic variables (age, birthplace, employment status, marital status, and health insurance coverage) and physiological and behavioral variables (smoking and alcohol consumption). *P < 0.05.

Of PLWH aged 18–49 years, males had a significantly higher predicted marginal probability than females for decreased HDL-C (65.6% vs. 48.5%), overweight/obesity (17.7% vs. 11.2%), DM (3.0% vs. 1.2%), and declined eGFR (13.8% vs. 8.4%). Of PLWH aged ≥50 years, the predicted marginal probability of having low CD4 counts was 46.8% for males compared to 35.5% for females.

### Prevalence of high CVD risk across age groups and sex differences

**Figure 2:**
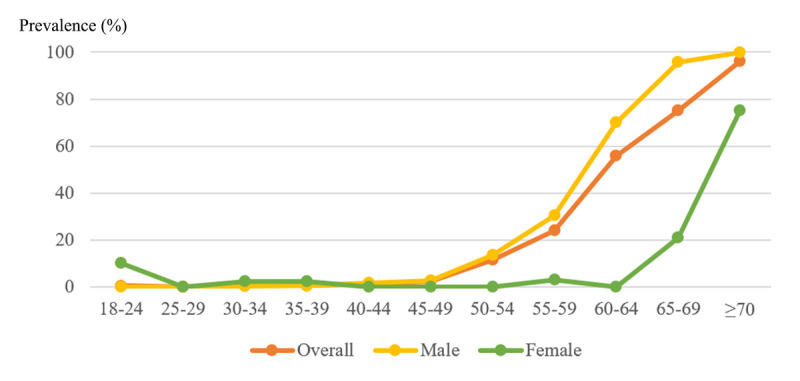
Prevalence of high cardiovascular disease risk among people living with HIV across age groups, stratified by sex.

The prevalence of high CVD risk increased sharply with age for both sexes. Breakpoint analysis indicated a steeper rise beginning at age 50 for men and around age 65 for women (Figure 2 and Supplementary Table S4). Using 10-year age groups yielded a similar pattern, with CVD risk beginning to rise around age 50 in men and around age 60 in women, consistent with the main analysis (Supplementary Figure S1).

## Discussion

Significant sex disparities were observed in the CVD risk and risk factors. Men living with HIV had a significantly higher predicted probability of high CVD risk compared with women. Among PLWH aged 18–49 years, men were significantly more likely to exhibit several CVD risk factors, including decreased HDL-C, overweight/obesity, DM, and declined eGFR. Additionally, men experienced a significant increase in CVD risk at an earlier age, whereas women showed a delayed but steeper increase in CVD risk. These findings highlight distinct sex-specific patterns in CVD risk accumulation among PLWH.

We found that dyslipidemia was the most prevalent CVD risk factor among PLWH, with a particularly high prevalence of low HDL-C. Notably, men under 50 years old were significantly more likely to have decreased HDL-C than women. The prevalence of low HDL-C among PLWH was substantially higher than that of the general population (66.7% vs. 20.4%) (16), which aligns with previous studies (17,18). This may be attributed to the Nef protein shed by HIV, which can stimulate cholesterol biosynthesis and inhibit its efflux, while simultaneously disrupting caveolin-dependent cholesterol transport in infected macrophages, resulting in decreased HDL-C levels (19). Additionally, ART regimens and their duration were associated with HDL-C level (19,20,21). The higher likelihood of decreased HDL-C in men has also been observed in previous research (22). It partly due to the protective effects of endogenous estrogen in premenopausal women, which promotes higher HDL-C levels and more favorable lipid metabolism (23,24). Therefore, regular monitoring of lipid profiles, particularly HDL-C levels, should be an integral part of standard care for PLWH, with special attention given to men who are at higher risk for dyslipidemia. For patients with persistently low HDL-C levels, clinicians should assess potential ART non-adherence and, if necessary, consider switching to an alternative regimen (20). This proactive approach could help mitigate cardiovascular risk in this vulnerable population. Additionally, ensuring equitable access to regular lipid monitoring, ART adherence support, and regimen adjustment options is particularly important for socially vulnerable subgroups within the PLWH population, who may face structural barriers in accessing comprehensive cardiovascular care.

Men living with HIV exhibit a significantly higher prevalence of multiple CVD risk factors compared to women of young age, including overweight/obesity, DM, and declined eGFR. This is consistent with observations in the general population, where men present a worse risk factor profile than women (25). This disparity may be attributed to both biological sex differences and behavioral practices. Human lipidomic profiles exhibit inherent sex-specific differences, driven by hormonal and metabolic pathways, that lead men to experience an increase in LDL-C levels and a decrease in HDL-C levels from early adulthood to middle age, while women typically maintain lower LDL-C and higher HDL-C levels (26). This lipid profile for men is more atherogenic and predisposes them to metabolic disturbances and DM. Moreover, our results showed that high-risk behaviors, such as smoking and alcohol consumption, were predominantly observed in men, with very few occurrences among women. In addition, women may have healthier dietary profiles, characterized by higher consumption of fruits and vegetables (27). Women are also more likely to be concerned about weight control and the naturalness of their food. These sex differences in risk factors may contribute to the increased likelihood of early adverse CVD events in men and lower risk in women. Healthcare providers should focus on early monitoring and targeted management of these risk factors in men from a younger age. Regular monitoring of blood glucose and renal function indicators, along with early lifestyle interventions, should be prioritized to promptly address CVD risk. Given that one-third of CVD occurrences among PLWH were attributable to smoking (28), smoking cessation is strongly recommended in clinical practice. Policies should promote access to counseling services, nicotine replacement therapies, and ongoing support to ensure long-term cessation success, ultimately contributing to a reduction in CVD burden.

The prevalence of high CVD risk increases with age and exhibits a distinct breakpoint, occurring at age 65 for women, which is significantly later than the age 50 breakpoint observed in men. This delay is likely partly due to the loss of endogenous estrogens during menopause, as higher levels of endogenous estrogen are thought to have a protective effect on the heart (29,30). Additionally, some lipid species show distinct and opposite age-related trajectories in men and women (31). Higher levels of sphingomyelin in older women compared to men suggest a potential sex-specific dysregulation of sphingolipid metabolism with age, which may contribute to the increased CVD risk in older women (31). However, this delayed onset of CVD in women should not lead to the misconception that women are “protected” against CVD. Although women may benefit from a natural delay in the onset of CVD compared to men, their lifetime risk and the number of cardiovascular events does not appear to be advantageous (32). The majority of CVD risk calculators did not consider female-specific risk factors, such as age of menarche and menopause, polycystic ovary syndrome, infertility and the use of assisted reproductive technology, spontaneous pregnancy loss, parity, adverse pregnancy outcomes, and female-predominant conditions, that can enhance CVD risk across a woman’s lifespan (29). This oversight may contribute to inequities in CVD risk prediction between sexes. Further efforts are needed to incorporate sex-specific risk factors and biomarkers into the current risk algorithms. Given that traditional risk factors confer a greater relative risk for CVD in women living with HIV compared to men, it is crucial to intervene promptly at the first sign of any CVD risk factor for women, even in low- or borderline risk stages. It’s essential to implement women-specific clinical guidelines to improve cardiovascular outcomes in women living with HIV. Additionally, efforts are also needed to understand whether, and if so how, these sex differences contribute to the sex differences in CVD among PLWH. Additionally, structural barriers, such as limited healthcare access, social stigma, and lower health literacy among some groups of women, must be addressed to ensure that these guidelines translate into improved outcomes for all female PLWH, regardless of their socioeconomic background.

This study provides new insights into sex-specific cardiovascular risk patterns among PLWH in China. By identifying earlier and more pronounced clustering of traditional risk factors in men, as well as a delayed but steeper increase in CVD risk among older women, our findings highlight the need for sex-tailored prevention strategies within HIV care. Clinically, these results support earlier and more intensive screening and lifestyle interventions targeting modifiable risk factors for men starting in young adulthood. It is crucial to heighten surveillance for women during and after the menopausal transition. Prompt intervention at the first sign of CVD is essential even when risk appears low or borderline. Furthermore, the observed sex disparities underline the limitations of existing risk prediction tools for PLWH, reinforcing the need to develop or refine models that incorporate female-specific factors. Integrating these findings into clinical practice may contribute to more equitable and effective cardiovascular risk management among PLWH.

## Limitations

There are several limitations in our study. First, information on ART regimens and certain social determinants of health, such as income, was unavailable in our EHR data. We were unable to analyze the potential impact of these factors on the outcomes of interest. Second, all participants were receiving HIV medical care, which may limit the generalizability of our findings to individuals who are not currently engaged in care. Third, although the study included all women hospitalized in the past 5 years, the proportion of women was relatively low, which may affect the robustness of gender-based analyses. Fourth, because our EHR data captures only hospitalized PLWH, whose health status may differ from those managed in outpatient settings, the generalizability of our findings to all PLWH should be interpreted with caution. Finally, the PCE was developed in Western populations and has not been validated in Chinese PLWH. Therefore, the absolute 10-year CVD risk may be under- or overestimated and should be interpreted with caution.

## Conclusion

Our study highlights significant sex differences in CVD risk and associated factors among Chinese PLWH, emphasizing the need for equity, and sex-specific strategies in cardiovascular care. Males under 50 years of age exhibited a higher prevalence of several modifiable CVD risk factors, calling for regular monitoring and targeted management from a younger age. On the other hand, while females showed a later onset of high CVD risk, the impact of menopause and associated changes in lipid metabolism underscores the importance of early intervention. Meanwhile, further efforts are needed to integrate female-specific factors into risk assessment tools to avoid under-recognition and delayed intervention in women living with HIV. To achieve cardiovascular health equity, it is critical to ensure that both men and women living with HIV have timely access to appropriate risk assessment, preventive care, and tailored interventions, regardless of age, gender, or socioeconomic status. Future research should explore not only biological mechanisms but also structural and systemic factors contributing to sex disparities in cardiovascular outcomes to guide precise and equitable prevention and treatment strategies.

## Additional File

The additional file for this article can be found as follows:

10.5334/gh.1553.s1 Supplementary Files. Tables S1 to S4 and Figure S1.
