# Supplementary material for: Sex Differences in Cardiovascular Disease Risk and Associated Risk Factors among People Living with HIV in China
Source: Glob Heart. 2026 Apr 24;21(1):35. doi: 10.5334/gh.1553 (PMC13109912; doi:10.5334/gh.1553)
Supplement: Supplementary Files. — Tables S1 to S4 and Figure S1. [file gh-21-1-1553-s1.pdf]

Table S1 Sex-by–risk factor interaction analyses for high predicted CVD risk among people living with HIV

	OR	***95% CI***	***P* value**
TC	0.565	(0.118-2.714)	0.476
HDL-C	1.204	(0.295-4.904)	0.796
LDL-C	0.375	(0.067-2.085)	0.262
TG	0.981	(0.238-4.049)	0.979
BMI	1.676	(0.325-8.641)	0.537
DM	4.363	(0.769-24.737)	0.096
SBP	0.480	(0.096-2.408)	0.372
eGFR	1.314	(0.328-5.268)	0.700
HIV-RNA	0.781	(0.195-3.131)	0.728
CD4 count	0.499	(0.122-2.037)	0.333

Abbreviation: DM: diabetes mellitus, eGFR: estimated glomerular filtration rate, HDL-C: high-density lipoprotein cholesterol, LDL-C: low-density lipoprotein cholesterol, SBP: systolic blood pressure, TC: total cholesterol, TG: triglyceride

Table S2 Sex-stratified associations between cardiometabolic risk factors and high CVD risk among people living with HIV

	Female			Male		
	OR	***95% CI***	***P* value**	***OR***	***95% CI***	***P* value**
TC	4.468	(1.090-18.318)	0.038	2.751	(1.458-5.190)	0.002
HDL-C	1.606	(0.475-5.425)	0.446	2.351	(1.435-3.853)	0.001
LDL-C	3.109	(0.699-13.820)	0.136	2.134	(1.059-4.301)	0.034
TG	2.032	(0.605-6.826)	0.251	2.701	(1.690-4.315)	<0.001
BMI	1.165	(0.259-5.235)	0.842	1.936	(1.146-3.271)	0.014
DM	3.488	(0.724-16.806)	0.119	16.492	(8.375-32.475)	<0.001
SBP	6.804	(1.617-28.627)	0.009	4.695	(2.463-8.948)	<0.001
eGFR	1.975	(0.530-7.355)	0.310	1.464	(0.932-2.299)	0.098
HIV-RNA	1.430	(0.418-4.891)	0.569	1.425	(0.904-2.248)	0.128
CD4 count	1.200	(0.339-4.249)	0.778	0.814	(0.522-1.271)	0.365

Abbreviation: DM: diabetes mellitus, eGFR: estimated glomerular filtration rate, HDL-C: high-density lipoprotein cholesterol, LDL-C: low-density lipoprotein cholesterol, SBP: systolic blood pressure, TC: total cholesterol, TG: triglyceride

Table S3 Sex differences in CVD risk factors

	Logistic regression		Predicted marginal probability (%)			
	OR (95% CI)	***P* value**	**Female**	**Male**	**Prevalence difference**	***P* value**
**Model 1**						
**Age 18-49 years (n=2444)**						
High CVD risk	0.510 (0.148-1.754)	0.285	1.5 (-0.2-3.1)	0.8 (0.4-1.1)	-0.7	0.407
High TC	0.556 (0.367-0.841)	0.005*	14.8 (9.9-19.7)	8.8 (7.6-10.0)	-6.0	0.019*
Low HDL-C	2.176 (1.630-2.906)	<0.001*	46.8 (39.9-53.7)	65.7 (63.7-67.7)	18.9	<0.001*
High LDL-C	0.671 (0.420-1.073)	0.096	10.8 (6.6-15.1)	7.5 (6.4-8.6)	-3.3	0.143
High TG	0.997 (0.735-1.351)	0.983	33.5 (27.0-40.0)	33.4 (31.5-35.4)	-0.1	0.983
Overweight/obesity	2.111 (1.300-3.426)	0.003*	9.4 (5.4-13.4)	17.9 (16.3-19.5)	8.5	<0.001*
DM	2.023 (0.630-6.492)	0.236	1.5 (-0.2-3.1)	2.9 (2.2-3.6)	1.5	0.110
SBP elevated	1.095 (0.697-1.720)	0.695	11.3 (7.0-15.7)	12.3 (10.9-13.6)	0.9	0.686
Declined eGFR	1.360 (0.852-2.171)	0.197	10.3 (6.2-14.5)	13.6 (12.1-15.0)	3.2	0.154
HIV-RNA≥500 copies/ml	0.961 (0.714-1.292)	0.791	37.9 (31.3-44.6)	37.0 (35.0-39.0)	-0.9	0.792
CD4 count <200 cells/μL	0.939 (0.702-1.255)	0.671	42.9 (36.0-49.7)	41.3 (39.3-43.4)	-1.5	0.672
**Age ≥50 years (n=576)**						
High CVD risk	7.339 (3.838-14.034)	<0.001*	9.6 (4.2-15.1)	43.9 (39.4-48.5)	34.3	<0.001*
High TC	0.662 (0.375-1.168)	0.155	16.7 (9.8-23.5)	11.7 (8.8-14.6)	-5.0	0.190
Low HDL-C	1.671 (1.106-2.524)	0.015*	50.0 (40.8-59.2)	62.6 (58.1-67.0)	12.6	0.016
High LDL-C	0.838 (0.436-1.613)	0.598	11.4 (5.6-17.2)	9.7 (7.0-12.4)	-1.7	0.612
High TG	0.891 (0.582-1.365)	0.596	36.8 (28.0-45.7)	34.2 (29.9-38.5)	-2.6	0.599
Overweight/obesity	0.895 (0.539-1.484)	0.667	21.1 (13.6-28.5)	19.3 (15.7-22.9)	-1.8	0.673
DM	0.910 (0.494-1.677)	0.763	13.2 (7.0-19.4)	12.1 (9.1-15.1)	-1.0	0.768
SBP elevated	0.747 (0.402-1.391)	0.358	13.2 (7.0-19.4)	10.2 (7.4-12.9)	-3.0	0.389
Declined eGFR	0.902 (0.598-1.361)	0.623	46.5 (37.3-55.6)	43.9 (39.4-48.5)	-2.6	0.624

HIV-RNA≥500 copies/ml	1.035 (0.678-1.579)	0.874	37.7 (28.8-46.6)	38.5 (34.1-43.0)	0.8	0.873
CD4 count <200 cells/μL	1.624 (1.061-2.487)	0.026	35.1 (26.3-43.8)	46.8 (42.2-51.3)	11.7	0.021
**Model 2**						
**Age 18-49 years (n=2444)**						
High CVD risk	0.650 (0.177-2.383)	0.516	1.2 (-0.2-2.6)	0.8 (0.4-1.2)	-0.4	0.578
High TC	0.617 (0.394-0.967)	0.035*	13.5 (8.7-18.3)	8.9 (7.7-10.0)	-4.7	0.067
Low HDL-C	2.527 (1.852-3.449)	<0.001*	43.6 (36.5-50.8)	65.9 (64.0-67.9)	22.3	<0.001*
High LDL-C	0.664 (0.400-1.105)	0.115	10.9 (6.3-15.5)	7.5 (6.4-8.6)	-3.3	0.167
High TG	1.151 (0.831-1.594)	0.397	30.7 (24.3-37.1)	33.7 (31.7-35.6)	3.0	0.387
Overweight/obesity	2.055 (1.239-3.409)	0.005*	9.8 (5.5-14.0)	17.8 (16.3-19.4)	8.1	<0.001*
DM	2.657 (0.801-8.807)	0.110	1.2 (-0.2-2.5)	3.0 (2.3-3.7)	1.9	0.018*
SBP elevated	0.980 (0.607-1.581)	0.933	12.4 (7.4-17.4)	12.2 (10.8-13.5)	-0.2	0.933
Declined eGFR	1.840 (1.120-3.023)	0.016*	8.3 (4.8-11.7)	13.9 (12.5-15.3)	5.6	0.004*
HIV-RNA≥500 copies/ml	1.229 (0.893-1.692)	0.205	32.9 (26.5-39.4)	37.5 (35.5-39.4)	4.5	0.193
CD4 count <200 cells/μL	1.157 (0.849-1.578)	0.356	38.3 (31.5-45.1)	41.7 (39.7-43.8)	3.4	0.350
**Age ≥50 years (n=576)**						
High CVD risk	44.009 (16.709-115.912)	<0.001*	11.4 (7.3-15.4)	44.8 (41.5-48.2)	33.5	<0.001*
High TC	0.733 (0.399-1.346)	0.317	15.6 (8.8-22.3)	12.0 (9.0-15.0)	-3.6	0.346
Low HDL-C	1.454 (0.941-2.249)	0.092	52.7 (43.3-62.2)	61.8 (57.3-66.2)	9.0	0.097
High LDL-C	0.809 (0.403-1.624)	0.551	11.7 (5.5-17.9)	9.7 (7.0-12.4)	-2.0	0.569
High TG	0.895 (0.570-1.407)	0.632	36.5 (27.3-45.7)	34.0 (29.6-38.4)	-2.5	0.635
Overweight/obesity	0.779 (0.453-1.342)	0.368	22.9 (14.8-31.0)	18.9 (15.4-22.5)	-4.0	0.387
DM	1.087 (0.566-2.090)	0.802	11.7 (5.9-17.5)	12.6 (9.5-15.6)	0.9	0.798
SBP elevated	0.633 (0.330-1.214)	0.169	14.8 (7.7-21.9)	9.9 (7.2-12.6)	-4.9	0.213
Declined eGFR	0.902 (0.574-1.418)	0.656	46.3 (37.2-55.4)	44.0 (39.6-48.4)	-2.3	0.657
HIV-RNA≥500 copies/ml	1.043 (0.664-1.639)	0.856	37.6 (28.5-46.7)	38.6 (34.1-43.0)	1.0	0.855

CD4 count <200 cells/μL	1.634 (1.043-2.560)	0.032	35.2 (26.1-44.3)	46.9 (42.3-51.5)	11.8	0.026*

Abbreviation: DM: diabetes mellitus, eGFR: estimated glomerular filtration rate, HDL-C: high-density lipoprotein cholesterol, LDL-C: low-density lipoprotein cholesterol, SBP: systolic blood pressure, TC: total cholesterol, TG: triglyceride

Model 1 was unadjusted model

Model 2 was adjusted for sociodemographic variables (age, birthplace, employment status, marital status, and health insurance coverage).

**P*<0.05

Table S4 Two-piecewise linear regression results for high CVD risk prevalence

	Before age breakpoint		After age breakpoint	
	**Coefficient (95% CI)**	***P* value**	**Coefficient (95% CI)**	***P* value**
Overall	2.56 (-3.13, 8.25)	0.329	7.77 (4.69, 10.85)	<0.001
Male	2.76 (-3.08, 8.59)	0.308	9.00 (5.84, 12.15)	<0.001
Female	0.58 (-1.19, 2.35)	0.47	6.75 (4.99, 8.52)	<0.001

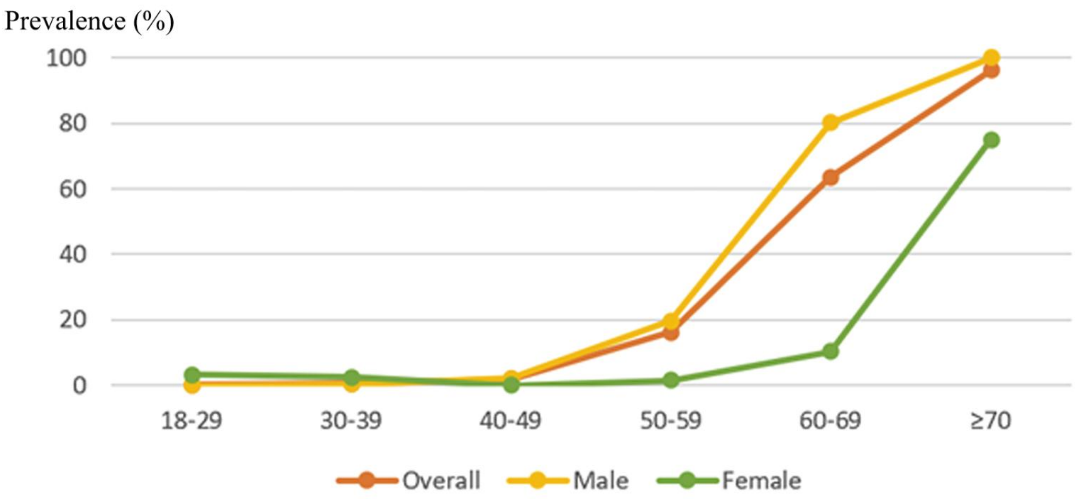

FigureS1 Age-specific prevalence of high cardiovascular disease risk by 10-year groups
